# Liver and Inflammatory Biomarkers Are Related to High Mortality in Hospitalized Patients with COVID-19 in Brazilian Amazon Region

**DOI:** 10.3390/life14070869

**Published:** 2024-07-11

**Authors:** Carla Sousa da Silva, Katrini Guidolini Martinelli, Marlison Wesley Miranda Viana, Deliane dos Santos Soares, Yasmin Garcia Silva Corrêa, Lucas Lima da Silva, Vanessa Salete de Paula, Luana Lorena Silva Rodrigues, Livia Melo Villar

**Affiliations:** 1 Programa de Pós-Graduação em Ciências da Saúde, Instituto de Saúde Coletiva, Universidade Federal do Oeste do Pará (UFOPA), Santarém 66075-110, Brazil; carlasousadasilva27@gmail.com (C.S.d.S.); luana.rodrigues@ufopa.edu.br (L.L.S.R.); 2 Laboratório de Hepatites Virais, Instituto Oswaldo Cruz (IOC/Fiocruz), Rio de Janeiro 21040-360, Brazil; lucaslima53@hotmail.com; 3 Departamento de Medicina Social, Universidade Federal do Espírito Santo (UFES), Vitória 29040-090, Brazil; katrigm@gmail.com; 4 Curso de Enfermagem, Universidade da Amazônia (UNAMA) Campus Santarém, Santarém 68010-200, Brazil; marlisonmiranda035@gmail.com; 5 Residência Multiprofissional em Estratégia Saúde da Família para as Populações do Baixo Amazonas, Instituto de Saúde Coletiva, Universidade Federal do Oeste do Pará (UFOPA), Santarém 66075-110, Brazil; delianedossantossoares@gmail.com; 6 Instituto de Biodiversidade e Florestas, Universidade Federal do Oeste do Pará (UFOPA), Santarém 66075-110, Brazil; yasmingarcia.scorrea@gmail.com; 7 Laboratório de Virologia e Parasitologia Molecular, Instituto Oswaldo Cruz (IOC/Fiocruz), Rio de Janeiro 21040-360, Brazil; vdepaula@ioc.fiocruz.br; 8 Laboratório de Aids e Imunologia Molecular, Instituto Oswaldo Cruz (IOC/Fiocruz), Rio de Janeiro 21040-360, Brazil

**Keywords:** biomarkers, COVID-19, SARS-CoV-2, liver, inflammation

## Abstract

COVID-19 is a multisystem disease with many clinical manifestations, including liver damage and inflammation. The objective of this study is to analyze inflammation biomarkers in relation to the clinical outcome and respiratory symptoms of COVID-19. This is a retrospective cohort of patients with COVID-19 admitted to the Hospital Regional do Baixo Amazonas from 2020 to 2022. Data were collected from electronic medical records from admission to the 30th day of hospitalization and soon after hospital discharge. A total of 397 patients were included in the study. In the longitudinal follow-up of liver markers, a significant difference was found for AST on day 14, with a higher median in the death group. Among the hematological markers, lymphopenia was observed throughout the follow-up, with the death group having the most altered values. When comparing the evolution of biomarkers in the Non-Invasive Ventilation (NIV) and Invasive Mechanical Ventilation (IMV) groups, AST showed a significant difference only on day 14 and GGT on day 1, being greater in the IMV group, and indirect bilirubin on day 7 being more altered in the NIV group. In conclusion, death during hospitalization or a more severe form of COVID-19 was related to significant changes in liver and inflammatory biomarkers.

## 1. Introduction

COVID-19, also known as coronavirus disease 2019, caused by the SARS-CoV-2 virus, is considered a systemic disease due to the involvement of multiple organs [1]. The mechanisms underlying multisystemic involvement may include liver damage and an exacerbated activation of the immune response, favoring disease progression [2]. This hypothesis was confirmed by laboratory alterations of liver biochemical markers, with elevation of the enzymes alanine aminotransferase (ALT) and aspartate aminotransferase (AST), increased bilirubin, gamma-glutamyl transferase (GGT) and alkaline phosphatase (AP) [3,4,5]. Among other inflammatory markers, whether biochemical or hematological, there is a greater potential for increased C-reactive protein (CRP), D-dimer (D-dimer), reduced lymphocyte count, Oxygen saturation (SaO_2_) and others [4,6]. The occurrence of liver dysfunctions reported during SARS-CoV-2 infection can be considered a possible sequelae of COVID-19, resulting from the worsening clinical condition [7,8]. The occurrence of liver damage during the period of SARS-CoV-2 infection has already been identified both in patients with a history of liver disease and patients without [3,4]. The main aggravating factor in SARS-CoV-2 infection stems from the dysfunction of the central role played by the liver in drug metabolism and central endogenous processes, such as coagulation, maintenance of osmotic pressure and production of acute phase reagents [9]. The loss of normal liver functions is directly related to in-hospital morbidity and mortality [8].

The longitudinal and dynamic evaluation of laboratory markers aids in knowing the relationship between the occurrence of liver damage and the inflammatory response triggered by the virus [6]. Furthermore, changes in biomarkers can be readily associated with diagnosis, prognosis, and outcomes. Some of these biomarkers are able to predict the severity of the disease, length of hospital stay at the intensive care unit (ICU) and death.

Therefore, the objective of this study is to analyze, over time, the inflammation biomarkers in relation to the clinical outcome (discharge and death), ventilatory mode (Non-Invasive and Invasive Mechanical) and city of origin (Santarém and other municipalities) of patients with COVID-19 in the Lower Amazon, Amazon, Brazil.

## 2. Materials and Methods

### 2.1. Study Design and Population

The study was carried out meeting all the criteria designated for research involving human beings established in Resolutions nº. 466/2012, 510/2016 and 580/2018; therefore, preserving the identity and ensuring the rights and duties of research participants. It was approved by the Ethics and Research Committee on 5 April 2022 with Opinion Number 5332611, CAAE 50826721.3.0000.0171. The Term of Free and Informed Consent was waived, as the study includes data collection from medical records, and the Trustee Term was used for institutional authorization.

This is a retrospective cohort study involving data from electronic medical records of patients diagnosed with COVID-19 who were hospitalized at the Regional Hospital of Baixo Amazonas (HRBA) from 25 March 2020 to 29 March 2022. The study time for each patient included was from admission to the hospitalization unit (day 1), followed by clinical follow-ups up to the 30th day of hospitalization and soon after hospital discharge. Patients who died or were discharged within a period prior to 30 days of hospitalization were also included in the study.

In order to obtain a representative number of samples, the total number of patients hospitalized for COVID-19 in the years 2020 and 2021 was investigated; in 2022 only hospitalizations until the month of March were considered. Given this information, the sample size was calculated using the formula for a finite population (<10,000 individuals). A sufficient sample was considered to compose the number of subjects: 397 medical records, proportionally divided into 132 from 2020, 236 from 2021 and 29 from 2022.

Inclusion criteria were availability of age information, at least one positive result from the real-time quantitative reverse transcription Polymerase Chain Reaction (qRT-PCR) test for SARS-CoV-2 from nasal and pharyngeal swab samples, carried out in accordance with the World Health Organization (WHO) protocol and stay at the hospital at least for seven days. Exclusion criteria were an absence of a positive molecular test (qRT-PCR) for SARS-CoV-2 after admission to the hospital and an absence of information in the medical record regarding laboratory tests for liver and inflammatory evaluation.

### 2.2. Data Collection

Data collection from the medical records involved six moments: the first was on day 1 (D1) of hospitalization, the moment of admission of the patient to the hospital, where all clinical, epidemiological and laboratory information was cataloged. The subsequent ones occurred on days 7 (D7), 14 (D14), 21 (D21) and 30 (D30) of hospitalization and after hospital discharge. The collection after discharge occurred from the multidisciplinary evolutions performed in the patient’s return visit to the hospital, held between 7 and 15 days after discharge.

The classification of liver injury presented by the patients was based on the evaluation in the medical reports, with an association of clinical signs and alterations in specific laboratory markers analyzed.

### 2.3. Statistical Analysis

A database was created for tabulating the variables. Initially, the data were analyzed descriptively, with absolute and relative frequency for the qualitative variables, and for the quantitative variables, due to their non-parametric distribution, the median with the 25th and 75th percentiles (P25–P75) was used.

Subsequently, for inferential analysis and to compare the biomarkers of liver alteration/injury and inflammation according to the groups, clinical outcome (discharge due to cure or death), ventilatory mode (Invasive Mechanical Ventilation and Non-Invasive Ventilation) and city of origin (Santarém and other cities), the Chi-square/Fisher’s Exact Test was used to compare qualitative independent variables and the Mann–Whitney test for quantitative independent variables with non-parametric distribution. An error of 5% and a confidence interval of 95% were used.

## 3. Results

### 3.1. Clinical and Epidemiological Profile

The sample of this study comprised 397 patients hospitalized for COVID-19 at the HRBA, of which 132 were hospitalized in 2020, 236 in 2021 and 29 in 2022 (Table 1). Most patients were male, aged 60 years or older, self-declared black skin color, had completed elementary school or incomplete high school, were married/stable union and came from the city of Santarém. When observing the hospitalization sector, it was noticed that 94.6% were hospitalized in the ICU and that the percentage of deaths exceeded the discharge outcome, affecting more than half of the cases (Table 1).

**Figure 1:**
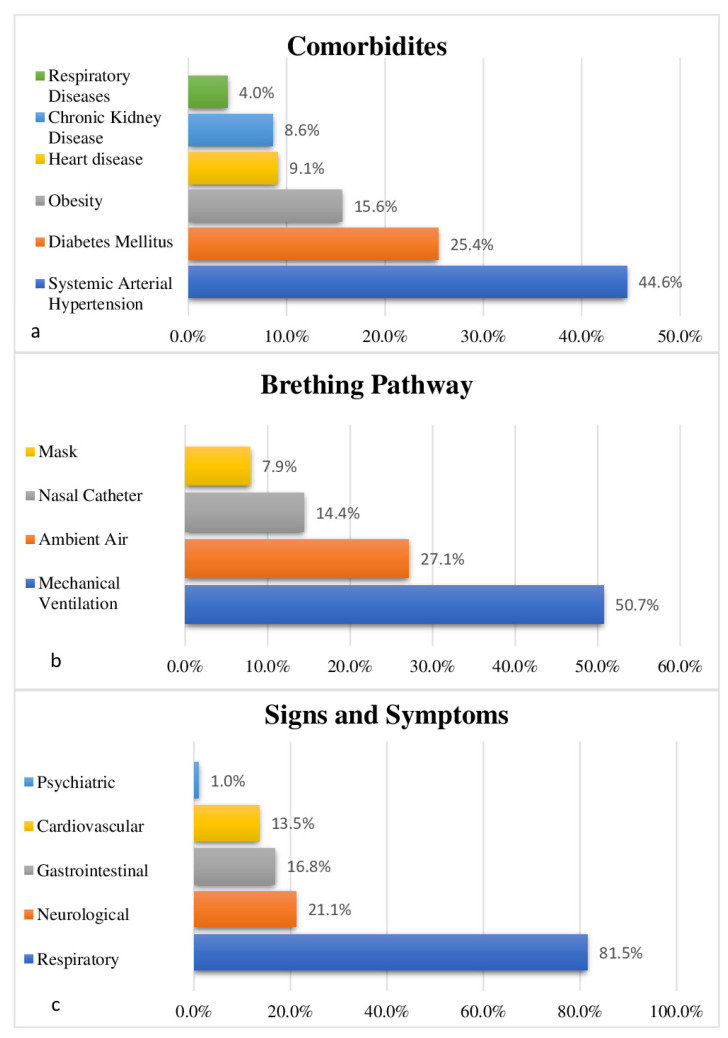
Clinical variables of patients with COVID-19 hospitalized between 25 March 2020 and 29 March 2022 at the Regional Hospital of Baixo Amazonas, Santarém-Pará, Brazil. Comorbidities (**a**). Signs and symptoms (**b**). Ventilation Support (**c**).

The analysis of clinical variables collected from patients upon hospital admission revealed that Systemic Arterial Hypertension (SAH), followed by Diabetes Mellitus (DM) and obesity, were the most reported comorbidities. When analyzing the signs and symptoms related to COVID-19 during hospitalization, a higher frequency of respiratory clinical manifestations was found, followed by neurological, gastrointestinal, cardiovascular and psychiatric manifestations. Regarding the breathing route, mechanical ventilation (MV) prevailed, followed by ambient air (Figure 1).

### 3.2. Liver Disorders and Inflammation

When analyzing the laboratory results of liver markers throughout the longitudinal study, D1, D7, D14, D21 and D30 days and after discharge, only bilirubin remained within normal parameters throughout the period of follow-up, while the other markers showed non-linear variations (Table 2). It is observed that AST, ALT and GGT are elevated in all time intervals, with more altered medians on D7 (52.0 U/L) for AST, on D1 (79.3 U/L) for ALT and on D7 (196.0 U/L) for GGT. The AP, on the other hand, is elevated from D7 to D30 and returned to normality after hospital discharge (Supplementary Table S1).

As for other inflammation markers, only SaO_2_ remains normal at all time intervals. On the other hand, CRP is the biomarker that shows higher values and is more distant from the reference standard, followed by D-dimer and lymphocyte count, which remains altered at almost all times during hospitalization, except shortly after hospital discharge (Table 3). The interval with the greatest change in these markers is on D21, with the highest increase in CRP (106.1 mg/dL) and on D7, with the greatest change in D-dimer (2431.0 ng/mL) and lymphocyte count (6.8/mm^3^) (Supplementary Table S1).

**Figure 2:**
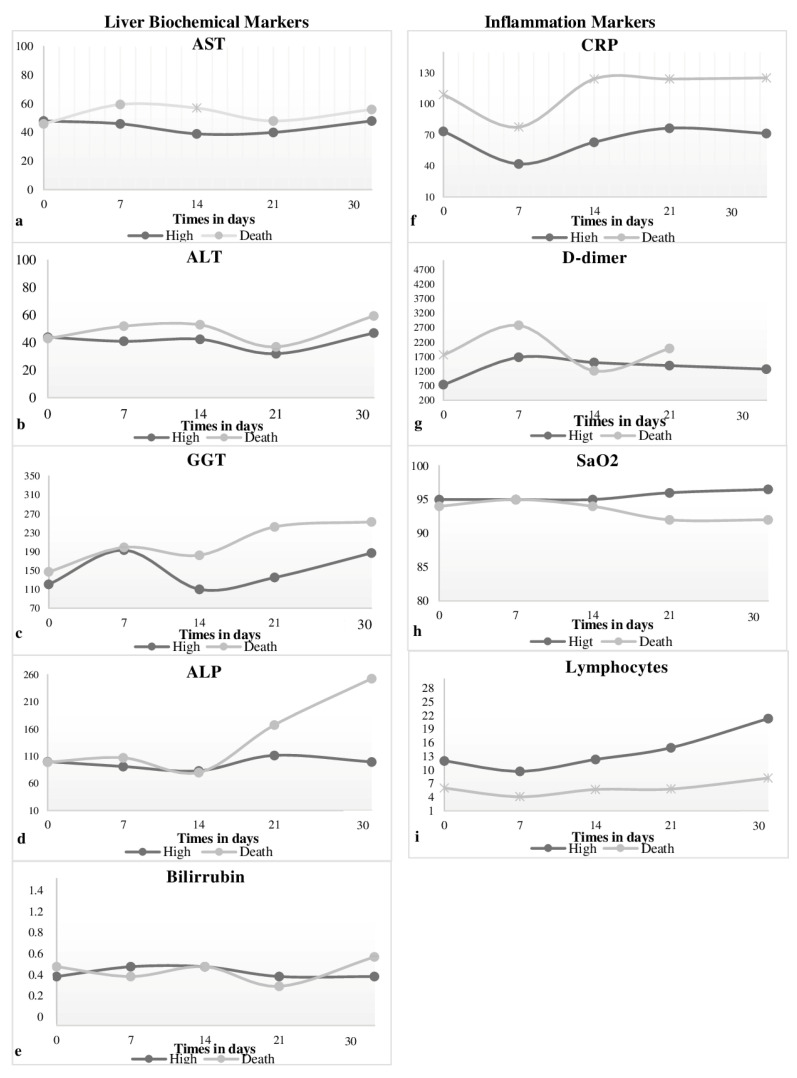
Curves exploratory assessments of rates of change in alteration/injury marker values hepatic: AST (**a**), ALT (**b**), GGT (**c**), AP (**d**) and bilirubin (**e**). Inflammation markers: CRP (**f**), D-dimer (**g**), SaO_2_ (**h**) and lymphocytes (**i**) by the median, using the Mann–Whitney test considering a *p*-value of 0.05 as meaningfulness statistic during the hospitalization period of the longitudinal study of patients hospitalized with COVID-19 between 2020 and 2022, at the Regional Hospital of Baixo Amazonas, Santarém-Pará, Brazil. * = *p*-value less than or equal to 0.05 using the Chi-square/Fisher’s Exact test.

When comparing the values of the results of the hepatic markers throughout the study longitudinally between the groups of discharge and death patients, a significant difference was found only on D14 (*p* = 0.031) for AST, with a higher median for the death group (57.0 U/L) when compared with the discharge group (39.0 U/L). CRP, D-dimer and lymphocyte count demonstrated high levels in the death group compared with the discharge group. When each biomarker was analyzed, it was found that there were significant differences on D1 (*p* = 0.001) for D-dimer, with a higher median for the death group (1759.0 ng/mL), and on D1 (*p* = 0.001, *p* = 0.000), D7 (*p* = 0.017, *p* = 0.000), D14 (*p* = 0.000, *p* = 0.000), D21 (*p* = 0.002, *p* = 0.000) and D30 (*p* = 0.001, *p* = 0.000) for CRP and counting of lymphocytes (Figure 2).

### 3.3. Liver Injury and Mortality

The comparative longitudinal assessment of the levels of liver markers and inflammation markers categorized as low, normal or high between the two groups of patients, discharge and death, was only possible during the study hospitalization period, D1, D7, D14, D21 and D30 days, and showed a significant difference only for AST/TGO on D14 (*p* = 0.052). For the other liver markers, there is no difference between the groups, although the GGT is shown to be higher, regardless of the group of patients assessed (Table 2).

As for the other inflammation markers, all four biomarkers show a significant difference at different times of hospitalization, being always more altered in the death group. CRP was higher with a significant difference on D1 and D7 (*p* = 0.056, *p* = 0.008); the D-dimer was higher with a significant difference only in D1 (*p* = 0.004). SaO_2_ was more reduced with a difference on D21 (*p* = 0.002); the lymphocyte count pattern showed a predominance of lymphopenia *p*-value less than 0.05 throughout the longitudinal study (Table 3).

### 3.4. Biomarkers in Relation to Ventilation Mode

By categorizing the liver biochemical markers and inflammation markers biomarkers as low, normal or discharge and comparing the evolution in the Non-Invasive Ventilation (NIV) and Invasive Mechanical Ventilation (IMV) groups during the study hospitalization period (D1–D30), it was observed that the AST showed a significant difference only on D14, being higher in the IMV group (*p* = 0.008). The GGT also showed a significant difference on D1 (*p* = 0.010), being higher in the IMV group. Indirect bilirubin showed a significant difference on D7 (*p* = 0.050) and was more altered in the NIV group. For ALP, there was no difference between groups (Table 4).

All inflammation makers showed a significant difference at different times of hospitalization, always being more altered in the IMV group. CRP was higher with a significant difference on D1 and D7 (*p* = 0.001, *p* = 0.010); D-dimer was higher, demonstrating a significant difference in D1 (*p* = 0.035); the lymphocyte count pattern showed lymphopenia from D1 to D14 (*p* = 0.000, *p* = 0.003; *p* = 0.000) and SaO_2_ showed no significant difference (Table 5).

## 4. Discussion

Patients with COVID-19 hospitalized at HRBA had their epidemiological, clinical and laboratory data collected, and until 9 July 2020, this was the only reference hospital in the care of patients who needed medium- and high-complexity care in the regions of health Baixo Amazonas and Tapajós—located in the west of the state of Pará—one of the most remote areas in the north of Brazil. Subsequently, the demands of the pandemic in these regions were shared with the Hospital Regional do Tapajós (HRT)—Itaituba, which was recently opened and had reduced service capacity and infrastructure.

The highest occurrence of COVID-19 cases in 2021 observed in this study agrees with the national and international epidemiological scenario for that period. According to other reports, most COVID patients were male [10,11]. In this study, the most evident age in patients was 60 years or older, which corroborates studies published in the same period [12,13]. In our cohort, the most prevalent comorbidities were hypertension, DM and obesity, in line with a large body of scientific evidence on profiles of patients hospitalized for COVID-19 [12,14,15].

Studies demonstrate that the mortality of COVID-19 is associated with social issues involving access to public health and environmental factors. The period of increase in cases was consistent with the number of patients collected in this study and the collapse of the health network in the northern region of Brazil, where this study was developed [16,17,18].

The prevalence of respiratory signs and symptoms in those hospitalized with COVID-19 favored the induction of a large number of patients to dependence on ventilatory support, which is in line with multiple findings in the literature about signs and symptoms of this disease [19,20].

Regarding the behavior of liver markers throughout this longitudinal study, it was possible to observe, through the medians, the constant elevation of AST and GGT above normal values at all follow-up times for ALT only on D1, D7 returning to D30 and ALP from D7 to D30, with bilirubin within the normal range. Findings in the literature refer to the diversity of frequency of elevation in the levels of liver markers among different studies [21,22,23,24,25]. It is reported that at the beginning of SARS-CoV-2 infection, there is an increase in transaminases first, followed by cholestatic enzymes, while other patients have increased serum bilirubin levels [26]. Those with severe and critical conditions are more likely to develop severe liver damage compared with mild and moderate cases [27,28,29]. In a cohort by Diaz-Louzão et al. (2022), levels of AST, ALT, GGT and AP altered in patients with COVID-19 showed an increase of 53%, 56%, 42% and 19%, respectively. Similar to our findings, other studies report that bilirubin during COVID-19 is normal or slightly increased, as well as AP and GGT may be increased in up to 50% of cases, indicating damage to cholangiocytes [21,23,24].

We identified throughout the longitudinal study that the elevation of AST was significantly associated with the clinical outcome of death and the IMV group on D14 equivalent to the fourteenth day of hospitalization. For the IMV group, a significant difference was also found for GGT on D1 and indirect bilirubin on D7 in the NIV group. It is believed that liver impairment in SARS-CoV-2 infection is associated with the worsening of COVID-19, increased length of stay, need for ventilatory support, and mortality. Studies show that AST is recurrently higher than ALT due to the multisystemic nature of COVID-19, and AST is present in other tissues and is less specific for the liver when compared with ALT. AP and total bilirubin may manifest in 20–30% and 4–16%, respectively, so that cholestasis appears on average in 15% of hospitalized patients, creating a pattern of liver damage. In most cases, liver manifestations are accompanied by changes in inflammation markers; however, it is not known whether liver changes are previously triggered by the virus or occur due to events within the disease [21,30].

Our findings demonstrate that COVID-19 patients are predisposed to manifest liver alteration/injury during hospitalization and even in a brief period soon after hospital discharge and that those who present more elevation of the biomarker AST are more likely to have a worse clinical outcome. Liver damage can generate dysregulated immune responses capable of causing cytokine storm, a pathological condition that can lead to the patient’s death. Therefore, liver and inflammation markers can help in the evaluation of the patient’s clinical condition, in addition to predicting the severity of the disease [31].

Regarding the behavior of makers throughout this longitudinal study, it was possible to analyze the constant changes, with the exception of SaO_2_, but with a significant association of elevated CRP and D-dimer and reduced lymphocyte count with the outcome of death and the IMV respiratory condition during hospitalization for COVID-19. Lymphopenia is described as part of the progression of COVID-19, being a late complication associated with the cytokine storm present in critically ill patients [32,33].

Finally, when observed throughout the longitudinal study, the curves that demonstrate the behaviors of markers simultaneously in the discharge and death groups show similar behaviors observed between them. Studies suggest that the systemic inflammatory response resulting from tissue hypoxia and drug use can cause liver damage/alteration in patients with COVID-19, allowing the visualization of disorders in these biomarkers [34,35].

The main limitations of the study include little information found after hospital discharge, which is linked to the low adherence of patients who survived the return hospital visit. None of the patients had a history of viral hepatitis B and C. The influence of multiple drug treatments during hospitalization, as well as inflammatory cytokines, was not evaluated. Patients with pre-existing liver disease may have been included in the study.

## 5. Conclusions

This study reinforces the existence of changes in liver alteration/injury and inflammatory biomarkers during the hospitalization of patients with COVID-19. This cohort revealed that mortality was higher for patients who had significant alterations in the markers of liver alteration/injury AST, GGT and bilirubin and in inflammatory biomarkers during hospitalization, although it was not possible to observe a sequential and linear correlation of the values exams over time. Patients on invasive mechanical ventilation had more changes in biomarkers of liver injury/alteration and inflammation than those on non-invasive ventilation.

## Figures and Tables

**Table 1:** Sociodemographic and epidemiological variables of patients with COVID-19 hospitalized between 25 March 2020 and 29 March 2022 at the Regional Hospital of Baixo Amazonas, Santarém-Pará, Brazil.

Variables	N (397)	(%)
Year		
2020	132	33.2
2021	236	59.4
2022	29	7.3
Sex		
Masculine	264	66.5
Feminine	133	33.5
Age (years) *		
0–19 years	14	3.6
20–39 years	57	14.5
40–59 years	120	30.6
60 years or older	201	51.3
Color/race *		
White	28	7.7
Black	333	91,2
Indigenous	4	1.1
Education *		
Illiterate/incomplete elementary school	7	3.0
Elementary complete/incomplete secondary	137	57.8
Full medium	66	27.8
Complete or incomplete higher education	27	11.4
Marital status *		
Single/widowed/separated	149	41.4
Married/stable union	211	58.6
City *		
Alenquer	14	3.6
Itaituba	11	2.8
Monte Alegre	26	6.7
Oriximiná	30	7.7
Prainha	6	1.5
Santarém	262	67.2
Others	41	10.5
Admission sector *		
Clinic	21	5.4
ICU **	369	94.6
Clinical outcome		
High	191	48.1
Death	206	51.9

* Values change according to data availability. ** ICU = intensive care unit.

**Table 2:** Comparative analysis of normal and abnormal values of markers of liver damage/alteration in relation to the clinical outcome of discharge and death using the Chi-square/Fisher’s Exact Test, considering a *p*-value of 0.05 as statistical significance, when longitudinal study of patients hospitalized for COVID-19 between 25 March 2020 and 29 March 2022 at the Regional Hospital of Baixo Amazonas, Santarém-Pará, Brazil.

		D1			D7			D14			D21			D30			ADH
		Discharge	Death		Discharge	Death		Discharge	Death		Discharge	Death		Discharge	Death		Discharge
Variable	Reference Value	N (%)	N (%)	*p*-Value	N (%)	N (%)	*p*-Value	N (%)	N (%)	*p*-Value	N (%)	N (%)	*p*-Value	N (%)	N (%)	*p*-Value	N (%)
Liver Biochemical Markers																	
AST/TGO	11 to 30 U/L			0.613			0.571			0.052			0.091			0.672	
Normal		51 (35.9)	63 (38.2)		23 (37.1)	20 (32.3)		28 (50.9)	10 (26.3)		19 (47.5)	4 (23.5)		12 (44.4)	2 (28.6)		18 (66.7)
Discharge		91 (64.1)	102 (61.8)		39 (62.9)	42 (67.7)		27 (49.1)	28 (73.7)		21 (52.5)	13 (76.5)		15 (55.6)	5 (71.4)		9 (33.3)
ALT/TGP	11 to 45 U/L			0.858			0.178			0.563			0.685			0.605	
Normal		74 (52.1)	85 (52.2)		34 (55.7)	23 (39.0)		27 (54.0)	16 (45.7)		21 (55.7)	10 (62.5)		10 (40.0)	3 (50.0)		17 (63)
Discharge		68 (47.9)	78 (47.9)		27 (44.3)	36 (61.0)		23 (46.0)	19 (54.3)		17 (44.7)	6 (37.5)		15 (60.0)	3 (50.0)		10 (37.0)
GGT	7 to 58 U/L			0.104			0.193			1.000			-			1.000	
Normal		10 (14.7)	5 (5.4)		2 (11.8)	0 (0.0)		1 (10.0)	0 (0.0)		0 (0.0)	0 (0.0)		1 (14.3)	0 (0.0)		1 (11.1)
Discharge		58 (85.3)	88 (94.6)		15 (88.2)	21 (100.0)		9 (90.0)	6 (100.0)		7 (100.0)	2 (100.0)		6 (85.7)	1 (100.0)		8 (88.9)
ALP	27 to 100 U/L			0.297			0.268			1.000			1.000			0.464	
Normal		38 (51.4)	49 (50.5)		8 (57.1)	8 (38.1)		5 (55.6)	3 (60.0)		3 (37.5)	1 (50.0)		3 (50.0)	0 (0.0)		3 (100.0)
Discharge		36 (48.6)	48 (49.5)		6 (42.9)	13 (61.9)		4 (44.4)	2 (40.0)		5 (62.5)	1 (50.0)		3 (50.0)	2 (100.0)		0 (0.0)
total bilirubin	≤1.2 mg/dL			0.173			0.096			0.695			1.000			1.000	
Normal		95 (88.0)	106 (81.5)		18 (69.2)	32 (86.5)		14 (66.7)	18 (72.0)		9 (81.8)	5 (83.3)		7 (87.5)	4 (80.0)		6 (75.0)
Discharge		13 (12.0)	24 (18.5)		8 (30.8)	5 (13.5)		7 (33.3)	7 (28.0)		2 (18.2)	1 (16.7)		1 (12.5)	1 (20.0)		2 (25.0)
Direct Bilirubin	≤1.2 mg/dL			0.239			0.231			0.357			1.000			0.385	
Normal		104 (95.4)	120 (91.6)		23 (85.2)	35 (94.6)		20 (95.2)	21 (84.0)		10 (90.9)	6 (100.0)		8 (100.0)	4 (80.0)		7 (87.5)
Discharge		5 (4.6)	11 (8.4)		4 (14.8)	2 (5.4)		1 (4.8)	4 (16.0)		1 (9.1)	0 (0.0)		0 (0.0)	1 (20.0)		1 (12.5)
Indirect Bilirubin	≤1.2 mg/dL			0.650			0.302			0.346			-			0.385	
Normal		104 (94.5)	122 (93.1)		24 (88.9)	36 (97.3)		19 (90.5)	24 (96.0)		11 (100.0)	6 (100.0)		8 (100.0)	4 (80.0)		7 (87.5)
Discharge		6 (5.5)	9 (6.9)		3 (11.1)	1 (2.7)		2 (9.5)	1 (4.0)		0 (0.0)	0 (0.0)		0 (0.0)	1 (20.0)		1 (12.5)

D1 = day one, D7 = day seven, D14 = day fourteen, D21 = day twenty-one, D30 = day thirty, ADH = after hospital discharge, N = number, % = percentage, AST/TGO = Oxaloacetic Transaminase, ALT/TGP = Glutamic Pyruvic Transaminase, GGT = Gamma Glutamyl Transferase, ALP = Alkaline Phosphatase. Chi-square/Fisher’s Exact Test.

**Table 3:** Comparative analysis of normal and abnormal values of markers of inflammation markers in relation to the clinical outcome of discharge and death using the Chi-square/Fisher’s Exact Test, considering a *p*-value of 0.05 as statistical significance, when longitudinal study of patients hospitalized for COVID-19 between 25 March 2020 and 29 March 2022 at the Regional Hospital of Baixo Amazonas, Santarém-Pará, Brazil.

		D1			D7			D14			D21			D30			ADH
		Discharge	Death		Discharge	Death		Discharge	Death		Discharge	Death		Discharge	Death		Discharge
Variable	Reference Value	N (%)	N (%)	*p*-Value	N (%)	N (%)	*p*-Value	N (%)	N (%)	*p*-Value	N (%)	N (%)	*p*-Value	N (%)	N (%)	*p*-Value	N (%)
Inflammation Markers																	
CRP	≤5 mg/dL			0.056			0.008			0.213			0.367			1.000	
Normal		9 (5.5)	3 (1.7)		10 (7.2)	1 (0.8)		5 (4.3)	1 (0.9)		4 (5.6)	1 (1.5)		1 (1.9)	0 (0.0)		8 (22.9)
Discharge		156 (94.5)	176 (98.3)		129 (92.8)	129 (99.2)		111 (95.7)	114 (99.1)		67 (94.4)	66 (98.5)		53 (98.1)	29 (100.0)		27 (77.1)
D-dimer	≤500 ng/mL			0.004			0.103			0.675			0.490			0.143	
Normal		40 (43.5)	28 (24.6)		6 (23.1)	1 (4.3)		6 (31.6)	2 (20.0)		1 (7.7)	1 (20.0)		0 (0.0)	1 (100.0)		4 (57.1)
Discharge		52 (56.5)	86 (75.4)		20 (76.9)	22 (95.7)		13 (68.4)	8 (80.0)		12 (92.3)	4 (80.0)		6 (100.0)	0 (0.0))		3 (42.9)
SaO_2_	90 to 99%			0.189			0.639			0.356			0.002			0.071	
Normal		122 (75.8)	131 (68.9)		95 (78.5)	114 (80.9)		78 (78.0)	90 (72.0)		58 (82.9)	43 (58.1)		34 (81.0)	20 (60.6)		11 (61.1)
Low		39 (24.2)	59 (31.1)		26 (21.5)	27 (19.1)		22 (22.0)	35 (28.0)		12 (17.1)	31 (41.9)		8 (19.0)	13 (39.4)		7 (38.9)
Lymphocytes	22 to 45/mm^3^			0.002			0.039			0.000			0.000			0.002	
Normal		34 (19.3)	14 (7.2)		19 (12.8)	7 (4.8)		27 (21.6)	3 (2.4)		22 (26.5)	2 (2.6)		24 (42.1)	3 (8.8)		24 (51.1)
Low		136 (77.3)	175 (89.7)		125 (83.9)	136 (93.2)		92 (73.6)	121 (96.0)		60 (72.3)	71 (93.4)		32 (56.1)	31 (91.2)		18 (38.3)
Discharge		6 (3.4)	6 (3.1)		5 (3.4)	3 (2.1)		6 (4.8)	2 (1.6)		1 (1.2)	3 (3.9)		1 (1.8)	0 (0.0)		5 (10.6)

D1 = day one, D7 = day seven, D14 = day fourteen, D21 = day twenty-one, D30 = day thirty, ADH = after hospital discharge, N = number, % = percentage, CRP = C-reactive protein, D-dimer = D-dimer, SaO_2_ = Oxygen saturation. Chi-square/Fisher’s Exact Test.

**Table 4:** Comparative analysis of the normal and altered values of the markers of alteration/liver injury in relation to the NIV and IMV using the Chi-square/Fisher’s Exact test, considering a *p*-value of 0.05 as statistical significance, during the longitudinal study, between 25 March 2020 and 29 March 2022 at the Regional Hospital of Baixo Amazonas, Santarém-Pará, Brazil.

		D1			D7			D14			D21			D30			ADH
		NIV	IMV		NIV	IMV		NIV	IMV		NIV	IMV		NIV	IMV		NIV
Variable	Reference Value	N (%)	N (%)	*p*-Value	N (%)	N (%)	*p*-Value	N (%)	N (%)	*p*-Value	N (%)	N (%)	*p*-Value	N (%)	N (%)	*p*-Value	N (%)
Liver Biochemical Markers																	
AST/TGO	11 to 30 U/L			0.545			0.444			0.008			0.425			0.868	
Normal		52 (38.0)	52 (35.4)		25 (38.5)	17 (30.4)		28 (56.0)	10 (24.4)		14 (46.7)	9 (34.6)		8 (40.0)	6 (42.9)		14 (66.7)
Discharge		85 (62.0)	95 (64.6)		40 (61.5)	39 (69.6)		22 (44.0)	31 (75.6)		16 (53.3)	17 (65.4)		12 (55.6)	8 (57.1)		7 (33.3)
ALT/TGP	11 to 45 U/L			0.516			0.239			0.649			0.135			0.160	
Normal		70 (50)	76 (53.5)		34 (53.1)	22 (41.5)		24 (52.2)	18 (0.0)		14 (48.2)	17 (70.8)		5 (27.8)	8 (61.5)		11 (52.4)
Discharge		70 (50.0)	66 (46.5)		30 (46.9)	31 (58.5)		22 (47.8)	19 (51.4)		15 (51.7)	7 (29.2)		13 (72.2)	5 (38.5)		10 (47.6)
GGT	7 to 58 U/L			0.010			0.486			0.464			-			0.571	
Normal		11 (18.0)	3 (3.0)		2 (10.5)	0 (0.0)		1 (10.0)	0 (0.0)		5 (100.0)	4 (100.0)		1 (14.3)	0 (0.0)		1 (12.5)
Discharge		50 (82.0)	85 (94.6)		11 (89.5)	18 (100.0)		9 (90.0)	5 (100.0)		0 (0.0)	0 (0.0)		6 (85.7)	2 (100.0)		7 (87.5)
ALP	27 to 100 U/L			0.883			1.000			0.872			0.065			0.673	
Normal		37 (54.4)	45 (50.5)		7 (43.8)	9 (47.4)		5 (55.6)	3 (60.0)		1 (16.7)	3 (75.0)		2 (33.3)	1 (50.0)		3 (100.0)
Discharge		31 (45.6)	44 (49.4)		9 (56.2)	10 (52.6)		4 (44.4)	2 (40.0)		5 (83.3)	1 (25.0)		4 (66.7)	1 (50.0)		0 (0.0)
Total Bilirubin	≤1.2 mg/dL			1.000			0.758			1.000			0.182			0.155	
Normal		87 (86.1)	103 (85.8)		23 (76.7)	27 (81.8)		16 (69.6)	16 (69.6)		7 (100)	7 (77.8)		5 (71.4)	6 (100.0)		4 (66.7)
Discharge		14 (13.9)	17 (14.2)		7 (23.3)	6 (18.2)		7 (30.4)	7 (30.4)		0 (0.0)	2 (22.2)		1 (28.6)	0 (0.0)		two ()
Direct Bilirubin	≤1.2 mg/dL			0.598			0.419			0.636			0.362			0.335	
Normal		94 (92.2)	114 (94.2)		27 (87.1)	31 (93.9)		20 (87.0)	21 (91.3)		7 (100.0)	8 (88.9)		6 (85.7)	6 (100.0)		5 (83.3)
Discharge		8 (7.8)	7 (5.8)		4 (12.9)	2 (6.1)		3 (13.0)	2 (8.7)		0 (0.0)	1 (11.1)		1 (14.3)	0 (0.0)		1 (16.7)
Indirect Bilirubin	≤1.2 mg/dL			0.419			0.050			0.073			-			0.335	
Normal		95 (92.2)	115 (95.0)		27 (87.1)	33 (100.0)		20 (87.0)	23 (100)		7 (100.0)	9 (100.0)		6 (85.7)	6 (100.0)		5 (83.3)
Discharge		8 (7.8)	6 (5.0)		4 (12.9)	0 (0.0)		3 (13.0)	0 (0.0)		0 (0.0)	0 (0.0)		1 (14.3)	0 (0.0)		1 (16.7)

NIV: Non-Invasive Ventilation; IMV: Invasive Mechanical Ventilation; D1 = day one, D7 = day seven, D14 = day fourteen, D21 = day twenty-one, D30 = day thirty, ADH = after hospital discharge, N = number, % = percentage, AST/TGO = Oxaloacetic Transaminase, ALT/TGP = Glutamic Pyruvic Transaminase, GGT = Gamma Glutamyl Transferase, ALP = Alkaline Phosphatase.

**Table 5:** Comparative analysis of normal and altered values of inflammation markers in relation to the NIV and IMV using the Chi-square/Fisher’s Exact Test, considering a *p*-value of 0.05 as statistical significance, during the longitudinal study, between 25 March 2020 and 29 March 2022 at the Regional Hospital from Baixo Amazonas, Santarém-Pará, Brazil.

		D1			D7			D14			D21			D30			ADH
		NIV	IMV		NIV	IMV		NIV	IMV		NIV	IMV		NIV	IMV		NIV
Variable	Reference Value	N (%)	N (%)	*p*-Value	N (%)	N (%)	*p*-Value	N (%)	N (%)	*p*-Value	N (%)	N (%)	*p*-Value	N (%)	N (%)	*p*-Value	N (%)
Inflammation Markers																	
CRP	≤ 5 mg/dL			0.001			0.010			0.058			0.184			0.284	
Normal		11 (6.8)	0 (0.0)		10 (7.6)	1 (0.8)		5 (4.9)	1 (0.8)		3 (5.2)	1 (1.3)		1 (2.6)	0 (0.0)		5 (20.8)
Discharge		150 (93.2)	176 (100.0)		122 (92.4)	131 (99.2)		98 (95.1)	123 (99.2)		55 (94.8)	77 (98.7)		37 (97.4)	43 (100.0)		19 (79.2)
D-dimer	≤ 500 ng/mL			0.035			0.080			0.334			0.929			0.212	
Normal		41 (41.8)	26 (26.8)		7 (21.9)	0 (4.3)		7 (33.3)	1 (14.3)		1 (11.1)	1 (12.5)		0 (0.0)	1 (33.3)		4 (66.7)
Discharge		57 (58.2)	81 (73.2)		25 (78.1)	17 (100.0)		14 (66.7)	6 (85.7)		8 (88.9)	7 (87.5)		4 (100.0)	2 (66.7)		2 (33.3)
SaO_2_	90 to 99%			0.064			0.278			0.875			0.897			1.000	
Normal		118 (77.1)	118 (67.4)		86 (76.1)	119 (82.1)		67 (73.6)	98 (75.4)		38 (69.1)	61 (70.1)		21 (72.4)	32 (71.1)		5 (55.6)
Low		35 (22.9)	57 (32.6)		27 (23.9)	26 (17.9)		24 (26.4)	32 (24.6)		17 (30.9)	26 (29.9)		8 (27.6)	13 (28.9)		4 (44.4)
Lymphocytes	22 to 45/mm^3^			0.000			0.003			0.000			0.116			0.164	
Normal		33 (19.3)	12 (6.9)		21 (14.7)	5 (3.4)		26 (23.0)	4 (3.0)		15 (22.1)	9 (10.1)		15 (36.6)	11 (22.4)		16 (51.6)
Low		130 (76.0)	161 (92.0)		118 (82.5	138 (93.9)		81 (71.7)	128 (95.5)		52 (76.5)	78 (87.6)		25 (61.0)	38 (77.6)		11 (35.5)
Discharge		8 (4.7)	2 (1.1)		4 (2.8)	4 (2.7)		6 (5.3)	2 (1.5)		1 (1.5)	2 (2.2)		1 (2.4)	0 (0.0)		4 (12.9)

NIV: Non-Invasive Ventilation; IMV: Invasive Mechanical Ventilation; D1 = day one, D7 = day seven, D14 = day fourteen, D21 = day twenty-one, D30 = day thirty, ADH = after hospital discharge, N = number, % = percentage, IMs = inflammation markers, CRP = C-reactive protein, D-dimer = D-dimer, SaO_2_ = Oxygen saturation.

## Data Availability

Datasets used and/or analyzed are available from the corresponding author upon reasonable request.

## Supplementary Materials

The following supporting information can be downloaded at: https://www.mdpi.com/article/10.3390/life14070869/s1, Table S1: Description of the results of the liver alteration/injury and inflammation markers by the median and 25th and 75th percentiles throughout the longitudinal study of patients hospitalized for COVID-19 between 25 March 2020 and 29 March 2022 at the Regional Hospital of Baixo Amazonas, Santarém-Pará, Brazil.
