# Supplementary material for: Liver and Inflammatory Biomarkers Are Related to High Mortality in Hospitalized Patients with COVID-19 in Brazilian Amazon Region
Source: Life (Basel). 2024 Jul 11;14(7):869. doi: 10.3390/life14070869 (PMC11277996; doi:10.3390/life14070869)
Supplement: Supplementary file 1 [file life-14-00869-s001.zip › life-2712460-supplementary.pdf]

Additional files

Table S1. Description of the results of the liver alteration/injury and inflammation markers by the median and 25th and 75th percentiles throughout the longitudinal study of patients hospitalized for COVID-19 between March 25, 2020 and March 29, 2022 at the Regional Hospital of Baixo Amazonas, Santarém-Pará, Brazil.

**Variables**	**D1**			**D7**			**D14**			**D21**			**D30**			**ADH**		
	Total exams	md	P25-P75	Total exams	md	P25-P75	Total exams	md	P25-P75	Total exams	md	P25-P75	Total exams	md	P25-P75	Total exams	md	P25-P75
**Liver Biochemical Markers**																		
AST/TGO	307	47.0	34.0-77.0	124	52.0	33.2-98.2	93	45.0	32.5-69.0	57	42.0	30.0-66.5	34	51.5	26.7-82.7	27	33.0	22.0-42.0
ALT/TGP	305	79.3	24.0-82.0	120	49.0	25.2-102.7	85	44.0	25.0-75.5	54	35.0	20.0-70.2	31	47.0	29.0-91.0	27	31.0	14.0-56.0
GGT	161	144.0	92.0-265.0	38	196.0	107.2-286.5	16	127.3	92.2-236.7	9	135.0	101.0-221.5	11	187.0	95.0-341.0	9	87.0	69.5-258.0
ALP	171	100.0	70.0-144.0	35	104.0	67.0-143.0	14	81.5	65.5-150.0	10	111.5	67.2-158.0	8	159.0	83.7-254.5	3	61.0	54.0-61.0
total bilirubin	238	1.0	0.3-0.9	63	0.6	0.3-1.0	46	0.6	0.3-1.4	17	0.5	0.3-1.0	13	0.6	0.3-0.8	8	0.5	0.4-2.4
direct bilirubin	240	0.5	0.2-0.5	64	0.3	0.2-0.5	46	0.3	0.1-0.5	17	0.3	0.2-0.6	13	0.3	0.2-0.5	8	0.2	0.2-0.2
indirect bilirubin	241	0.7	0.1-0.4	64	0.3	0.1-0.5	46	0.2	0.1-0.5	17	0.2	0.1-0.4	13	0.2	0.1-0.3	8	0.2	0.2-0.4
**Inflammation Markers**																		
PCR	344	93.9	45.7-130.9	269	61.5	22.7-116.8	231	91.8	39.0-141.6	138	106.1	48.4-141.7	83	93.1	42.8-141.0	35	25.1	6.0-61.5
D-dimer	206	1,087.0	321.2-3,102.7	49	2,431.0	1,054.1-3,000.0	29	1,224.0	432.6-3,448.0	18	1,603.7	968.7-3,000.0	7	1,104.2	693.0-2,161.7	7	466.5	160.4-2,099.7
SaO_2	351	95.0	88.0-98.0	262	95.0	91.0-98.0	225	95.0	89.0-98.0	144	94.5	86.2-98.0	75	95.0	85.0-98.0	19	95.0	81.0-98.0
Lymphocytes	371	8.5	4,6-16.4	295	6.8	3,1-14.0	251	8.3	4,7-16.4	159	9.7	5.0-18.2	91	15.0	7.3-25.4	47	27.9	17.8-39.3

D1= day one, D7= day seven, D14= day fourteen, D21= day twenty-one, D30= day thirty, ADH= after hospital discharge, Md = median, AST/TGO=Oxalacetic Transaminase, ALT/TGP=Glutamic Pyruvic Transaminase, GGT=Gamma Glutamyl Transferase, ALP=Alkaline Phosphatase, PCR=C-Reactive Protein, D-dimer=D-dimer, SaO_2 =Oxygen Saturation; P25 = 25th percentile; P75 = 75th percentile. Reference values: AST/TGO = 11 to 30 U/L, ALT/TGP = 11 to 45 U/L, GGT = 7 to 58 U/L, Bilirubin = up to 1.2 mg/dL, ALP = 27 to 100 U/L, PCR = up to 5 mg/dL, D-dimer = less than or equal to 500 ng / mL, SaO_2 = 90 to 99%, Lymphocyte = 22 to 45/mm^3.
